# Operative Workflow from CT to 3D Printing of the Heart: Opportunities and Challenges

**DOI:** 10.3390/bioengineering8100130

**Published:** 2021-09-22

**Authors:** Michele Bertolini, Marco Rossoni, Giorgio Colombo

**Affiliations:** Department of Mechanical Engineering, Politecnico di Milano, 20156 Milano, Italy; marco.rossoni@polimi.it (M.R.); giorgio.colombo@polimi.it (G.C.)

**Keywords:** patient-specific modeling, segmentation, heart model, 3D printing, stereolithography

## Abstract

Medical images do not provide a natural visualization of 3D anatomical structures, while 3D digital models are able to solve this problem. Interesting applications based on these models can be found in the cardiovascular field. The generation of a good-quality anatomical model of the heart is one of the most complex tasks in this context. Its 3D representation has the potential to provide detailed spatial information concerning the heart’s structure, also offering the opportunity for further investigations if combined with additive manufacturing. When investigated, the adaption of printed models turned out to be beneficial in complex surgical procedure planning, for training, education and medical communication. In this paper, we will illustrate the difficulties that may be encountered in the workflow from a stack of Computed Tomography (CT) to the hand-held printed heart model. An important goal will consist in the realization of a heart model that can take into account real wall thickness variability. Stereolithography printing technology will be exploited with a commercial rigid resin. A flexible material will be tested too, but results will not be so satisfactory. As a preliminary validation of this kind of approach, print accuracy will be evaluated by directly comparing 3D scanner acquisitions to the original Standard Tessellation Language (STL) files.

## 1. Introduction

Medical images have gained more and more importance in recent years, offering the opportunity to obtain detailed representations of the interior of the human body in a fast and simple way [1]. Imaging techniques in cardiovascular surgery and interventional cardiology mainly include Computed Tomography (CT), Magnetic Resonance Imaging (MRI) and echocardiography [2]. Although these imaging modalities are nowadays essential, they fail to provide a detailed understanding of the cardiovascular anatomy because of their intrinsic bidimensional nature [2]. In this sense, virtual 3D reconstruction methods may be very helpful, compensating for this deficiency but also paving the way towards a broad range of innovative applications in the field.

Image segmentation is “the process of partitioning an image into several parts, where each of these parts is a collection of pixels (or voxels) corresponding to a particular structure” [3]. With this process, tomographic data are used to create 3D models of the patient’s anatomy. Once the anatomy has been segmented, however, it is not yet suitable to be employed because of the limitations of the segmentation process. These include, among others, the “partial volume effect” and motion and signal artifacts [4], which cause the segmentation results to often be characterized by irregular and flawed shapes. Thus, an intense and difficult phase involving manually fixing the segmented result is required and is longer the more complex the involved geometry is.

**Figure 1:**
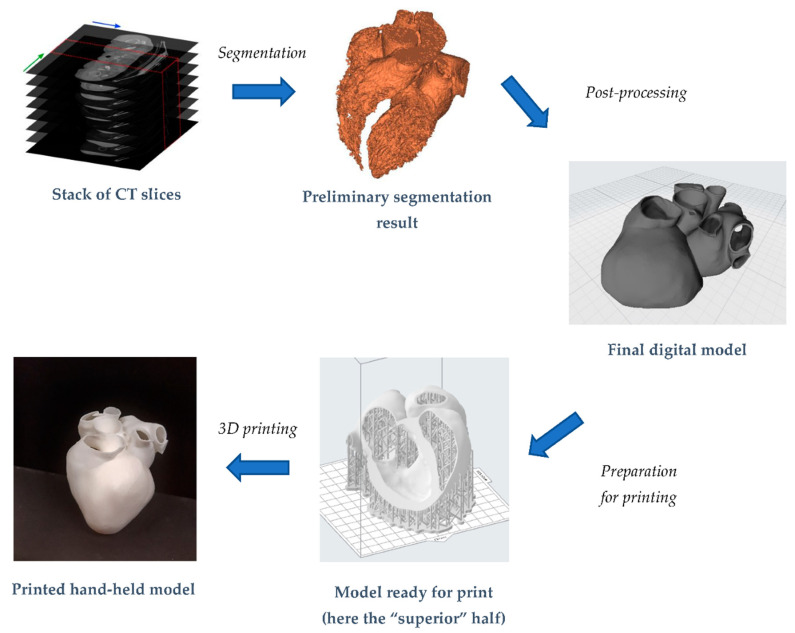
Key steps to obtain a hand-held heart model, from the stack of medical images until the final result.

In this paper, we will discuss in detail these steps focusing on the heart. This will help us to delineate the main criticalities in obtaining a digital patient-specific anatomical model. In the second part of the paper, the focus will be on the additive manufacturing process, through which different prints of heart models will be obtained, also varying the employed print material. We will present the challenges that we faced and the workflow through which we acquired the final hand-held models. Indeed, even if a general workflow is well-established (see Figure 1), there is not, to our knowledge, a paper which clearly and in detail retraces it, highlighting the operative problems that the user stumbles across.

This paper aims to offer a practical and methodological guide, operatively pointing out the workflow that, from a collection of CT scans, leads to the creation of tangible whole-organ models.

To add concreteness to the work, the dimensional accuracy of prints will be also evaluated. Following a reverse engineering approach, printed models will be reacquired; the obtained models will be then compared with the respective Standard Tessellation Language (STL) files. In this way, we will be able to gain an idea of how the digital and physical models match.

## 2. State of the Art

### 2.1. Reconstruction of an Anatomical District: Methods and Tools

A wide variety of segmentation techniques have been proposed in the literature [5]. Traditional segmentation algorithms use thresholding, region growing, edge detection and clustering, while, more recently, approaches based on deformable models and statistical, fuzzy and neural network techniques seem to be the major trend [6]. In general, rather than pursuing a general approach to segmentation, all these “new” algorithms target a specific application able to produce satisfactory results for a wide range of imaging applications. This is especially true when it comes to artificial intelligence-based segmentation. Given the outstanding performance in the field of image processing, Convolutional Neural Networks (CNNs) have been widely investigated in recent years [7]. However, in order for them to be trained properly, large datasets are necessary, leading each CNN to be very efficient only in the domain it was trained for. An extensive review of deep learning applied to cardiovascular system segmentation can be found in [8], where the authors also lamented the lack of generality of such approaches.

Manual segmentation is still the most diffused approach, even if semi-automatic and automatic tools have gradually been implemented [9]. Manual segmentation is a very time-consuming and tedious activity, subject to intra- and inter-observer variability, and requires dedicated expert operators [10]. Therefore, the implementation of automated segmentation approaches that could be accurate, robust and requiring as little user interaction as possible is perceived as a fundamental development in the field [9].

Currently, the processing of CT and MRI data is routinely performed with commercial software. Materialise Mimics (Leuven, Belgium) can be considered the state-of-the-art software in this field and is the most used by professionals worldwide [11]. However, usage of open-source software as an alternative to a commercial one is incrementally taking place because they guarantee satisfactory performance [11], are versatile and can be readily extended and redistributed. As main disadvantages, they often lack user-friendliness and are limited to research purposes.

### 2.2. Additive Manufacturing in the Cardiovascular Field: A Framework

Although three-dimensional printing (3DP) technology has been available for more than 30 years, only in the last ten years has it been introduced in the clinical arena [12]. Recently, 3DP technology has shown increasing use in many medical fields, from the creation of prosthetics, implants, fixtures and surgical tools to the reproduction of patient-specific 3D anatomical models [13]. A great advantage of this technology is the possibility of customization to obtain patient-specific models that can be used for personalized care.

Typically, the accuracy of a 3D printing object depends on the combination of the quality of medical images, the process for 3D modeling and the accuracy of the machine [14]. The 3DP technologies relevant to the biomedical field and cardiovascular applications include Fused Deposition Modeling (FDM), Stereolithography (SLA), Selective Laser Sintering (SLS) and material jetting. The pros and cons of each one, in terms of printable volume, costs and printing time, are well described in several papers [15,16]. In the following Table 1, we provide a systematic comparison of these printing techniques.

### 2.3. Additive Manufacturing in the Cardiovascular Field: Applications

Firstly, 3D printed models provide extra insight into the cardiovascular anatomy to complement the imaging data with regard to the position and size of the potential heart defects or to better appreciate relative positions among specific districts [17].

Even if the technology is not fully mature, nowadays, there are great potentialities in exploiting 3D-printed models in the cardiovascular field. We grouped them into three main applicative categories and analyzed them in detail: (1) 3DP as an educational tool, (2) 3DP for medical communication and (3) 3DP for surgery.

(1) Through 3D printing, accurate educational tools able to illustrate complex cardiovascular anatomy and pathology can be created [15]. Compared to 2D images, 3D renderings guarantee a better understanding of the human body and of fine anatomical details that may influence the management of the underlying disease [18]. This is especially true if we think about the complexity of the heart, above all in the presence of congenital heart diseases [19]. For this kind of application, the model is usually intended for visual inspection only, so the focus is on creating a high-resolution replica of the anatomy, while mechanical aspects are of secondary importance [18].
(2) Communication between cardiologists, cardiac surgeons and patients is very challenging, given also the complexity of medical terminology [20]. Therefore, the introduction of 3D-printed heart models during routine clinical consultations could be an appreciated improvement, as a preliminary study in the domain of congenital heart defects confirms [21].
(3) Moreover, 3D printing can be used to create and analyze models before starting actual surgery on the patient. The creation of high-fidelity training simulators for specific surgical procedures is also a possibility. Every patient’s anatomy is different, so surgeons’ practice on human cadavers, animal models and generic mannequins has often little relevance to the actual patient on the table. Decision-making in those cases considered complex and non-routine can surely benefit from the availability of physical 3D models, allowing an effective replication of surgical procedures, such as dissections, suturing or device sizing and placement (e.g., heart valves [22]), thus reducing operative risks [23] and operative room time. The employment of distensible resins in these cases surely helps to increase the realism and the reliability of the simulation. Indeed, here, differently from point (1), there is the need to carefully mimic the biomechanical properties of the involved tissue or organ, thus providing more realistic haptic feedback. The careful choice and characterization of materials become compulsory. As some studies suggest [24,25,26], when experimented, the adaption of 3DP has shown a reduction in procedure time and optimization of device deployment by improving the anticipation of potential obstacles in surgical procedures [27].

From an anatomical point of view, research applications of 3D printing in the cardiac field range from commonly reported structural heart diseases [28,29] to complex pediatric and adult congenital heart diseases [30,31] (e.g., atrial or ventricular septal defects). Additively manufactured models can regard the whole heart, but also specific portions of it, on the basis of the pathological region. An interesting review of potential applications of 3D printing for cardiac structures can be found in [32]. Focusing on structural heart diseases, the most relevant applications are related to the creation of patient-specific devices for closing the left atrial appendage [33,34] or for preparing aneurysmectomy [35]. Printed models to study valve pathologies, particularly of the aortic or mitral valves, are nowadays an object of great interest for simulations of procedures [36].

## 3. Digital Manufacturing of a Whole Heart Model

An introductory methodology overview for the work is now provided. The starting point is given by a stack of medical CT scans, included as the “data sample” within the segmentation software. From this, the heart was segmented, both as a blood pool and as a hollow model. The generated STL files were then exported in a post-processing environment to make them suitable for printing. Stereolithography technology was chosen because it guarantees very good accuracy at a reduced expense. Tests with both a rigid and a flexible resin were conducted. In dedicated software, models were properly oriented for printing and support structures were generated. Once prints were ready, they were first of all qualitatively evaluated. Then, by means of a laser 3D scanner, the correspondence between obtained point clouds and STL files was found and compared with data available in the literature.

### 3.1. Reconstruction of the 3D Digital Model

A stack of fully anonymized medical CT scans was used to generate the 3D model. It consisted of 393 slices in the axial plane, with a resolution of 512 × 512 pixels. The size of each pixel was a 0.35 mm square, while the slice thickness was 0.75 mm. Mimics software (Materialise, Leuven, Belgium, version 22) was used for segmentation.

**Figure 2:**
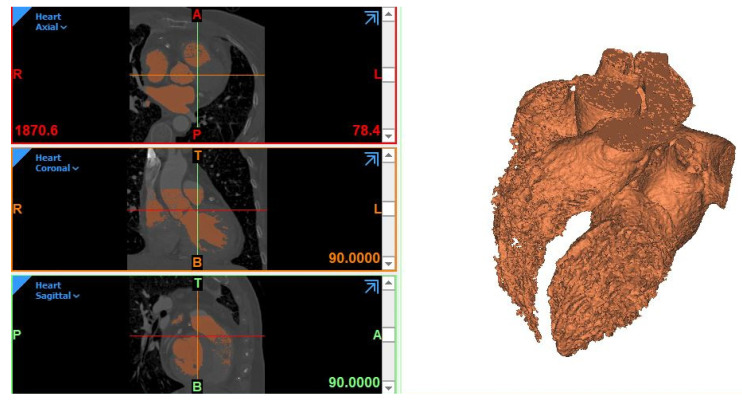
On the left, the segmented region seen according to three different planes from a Mimics screenshot. On the right, the correspondent preliminary, rough model of the blood pool. We can appreciate that the right ventricle is not well defined here.

It is important to preliminarily specify that, in the case of blood vessels or heart segmentation, the so-called “blood pool” is obtained: it represents only the fluid “inside” and not the surrounding tissues. This is the only region that we can isolate from CT scans, as the heart or vessel walls are mostly too thin and not detectable by the software in the segmentation process. The *Dynamic region growing* algorithm was first applied to the dataset in order to extract a mask. The overall selected threshold was between225 and15 Hounsfield Units (HU). After the specification of the Region of Interest (ROI) through the *crop mask* operation, manual editing (*Multiple slice edit* and *Edit mask* functions) was performed to guarantee a satisfactory correspondence between the generated mask and anatomical borders in the slices. A step of the work can be seen in Figure 2. We noticed that the right heart structures (right ventricle and atrium) were more difficult to be detected by the algorithm, thus resulting in a segmentation with lower quality with respect to the left heart structures (left ventricle and atrium and aorta). This was due to the lower amount of contrast that characterizes the right heart, which makes it more difficult for the algorithm to distinguish between the boundaries within the right heart and between the heart and surrounding voxels. Indeed, more attention and time were needed to fix the right heart segmentation in order to obtain a uniform-quality reconstruction.

After other minor corrections, the resulting 3D model was calculated from the mask. It was characterized by the presence of holes, frayed object parts, staircases and additional voxels. Thus, in order to obtain an exploitable geometry, it needed to be accurately post-processed, a phase that relies heavily on the expert clinical and anatomic knowledge of the graphic editor.

Thus, the model was exported to be post-processed in 3-Matic (Materialise, Leuven, Belgium, version 14). After a general remeshing, to achieve a uniform and denser tessellation, different operations were performed: first of all, a global smoothing step, in which a 1^st^-order Laplacian algorithm with shrinking compensation was used, followed by the manual local smoothing of areas still presenting relevant irregularities. This is a crucial step if we wish to achieve a continuous, uniform and smooth model. Smoothing intensity is up to the operator, even according to the final application intended for the model.

The anatomical accuracy required by a 3D cardiac model usually depends on its application. In our model, we decided to apply a quite high smoothing intensity. In this way, we could remove over-segmented details and mitigate the influence of irregular *trabeculae carnee* on the surfaces of the ventricles to obtain a more uniform and easily printable surface. Through specific tools, small holes still present from the initial segmentation were closed; moreover, some shells had to be removed and the mesh fixed (inverted normal, degenerated triangles, intersecting or overlapping ones and so on). In this way, a watertight mesh was obtained.

**Figure 3:**
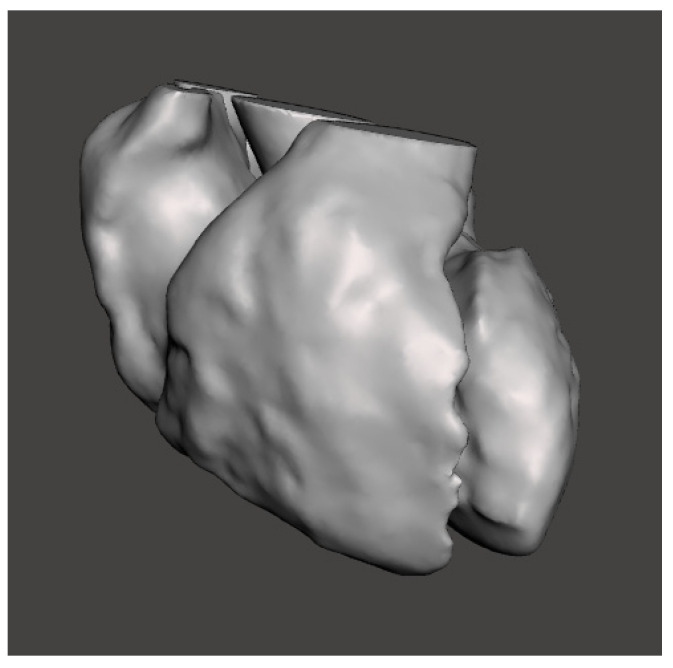
Blood pool model, as obtained after segmentation and post-processing.

Then, the blood pool model was ready to be exported as an STL file. The final model consisted of the 2 ventricles, the 2 atria and the first tract of the aorta, while other vessels were neglected. This model is shown in Figure 3.

Its surface mesh is composed of 66,998 triangles, with a surface area of around 443 cm^2^ and an occupied volume of around 337 cm^3^. At this stage, the 3D model, which can be considered as an “intermediate product”, is a full volumetric model, reflecting the space occupied by blood inside the heart.

**Figure 4:**
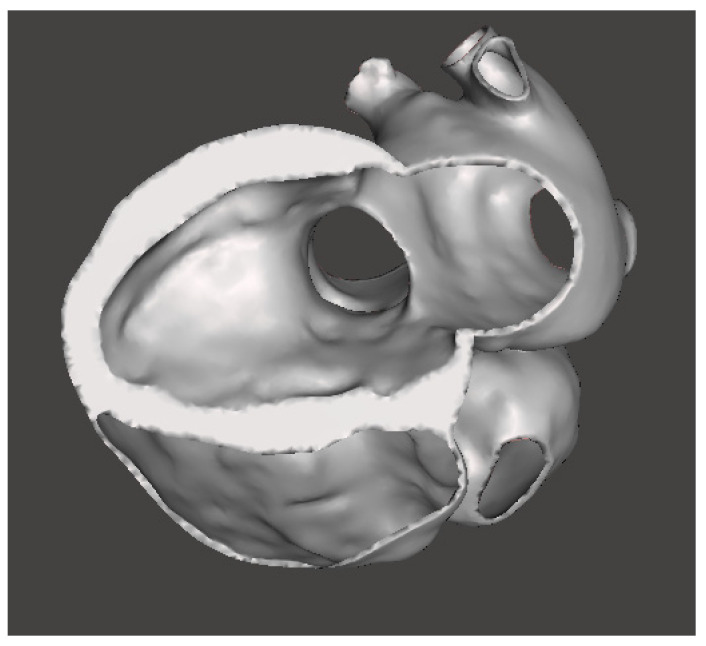
Section view of the reconstructed heart model, in which we can appreciate the realistic variable wall thickness.

A common technique used to produce a model of surrounding tissues is to generate the blood pool and then create a shell surrounding the model [37]. The shell can be adjusted to the desired thickness by extruding inwards, outwards or in both directions. Differently from some other works found in the literature (e.g., [31] or [38]), we did not want to consider the extruded wall thickness as constant, but we took into account its actual variability. Indeed, our solution was to segment the myocardium aside, from the same stack of CT images, and then, through Boolean operations between segmented results, obtain a more realistic complete heart shape. This is characterized by a wall thickness ranging from below 1 mm to more than 10 mm. In Figure 4, we can see the STL model (Supplementary materials, Heart_model.stl) in a section view, in which the variable profile of wall thickness can be appreciated.

In this case, the overall number of mesh triangles is equal to 103,076, the surface area becomes around 829 cm^2^, and the actual volume reduces to around 122 cm^3^.

These obtained STL models can be exploited for different engineering applications, including the realization of physical models through additive manufacturing, as illustrated below.

### 3.2. Heart Model 3D Print

For the printing phase, a Form 3 (Formlabs, Somerville, USA) machine was used. It is a desktop SLA printer able to create high-resolution objects (up to 25-microns resolution) at a relatively low cost. To prepare the model, *Preform* 3.3 (Formlabs, Somerville, MA, USA) software was used.

**Figure 5:**
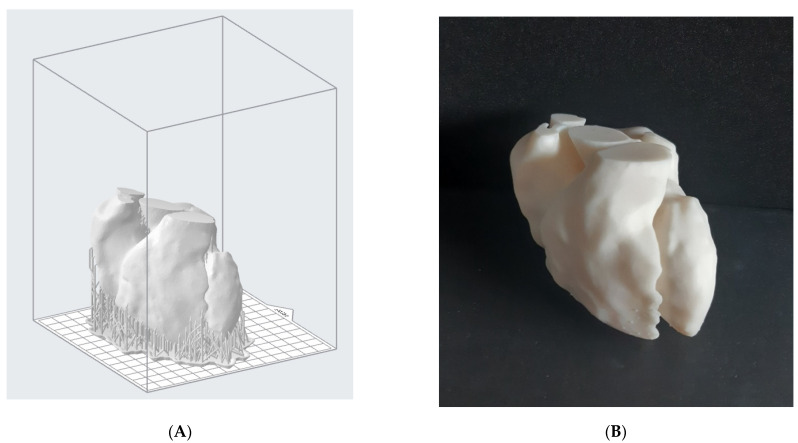
(**A**) Reconstructed digital model of the blood pool disposed onto the printing platform (Preform 3.3). We can appreciate the support structures that guarantee the printability of the model; (**B**) the corresponding printed result, after support removal and post-processing.

Firstly, we printed the blood pool model. After the STL file was imported into the software, it was necessary to properly orient the model on the printing platform; then, a support structure was generated, setting the support density and diameter. The layer thickness was set to 0.1 mm and a white standard Formlabs resin (V4), whose mechanical properties are shown in Table 2, was employed. The printing time was around 18 h, with a total number of 945 layers. The pre-processing step for the model is shown in Figure 5A.

Once the model was obtained, a post-processing treatment was needed. According to the material supplier, the object was washed in isopropyl alcohol for 20 min and then post-cured in the oven at 60 °C for 20 min. Supports were removed by means of flush cutters and the surface smoothed with sandpaper in order to eliminate marks left by the support structures. The result is shown in Figure 5B.

**Figure 6:**
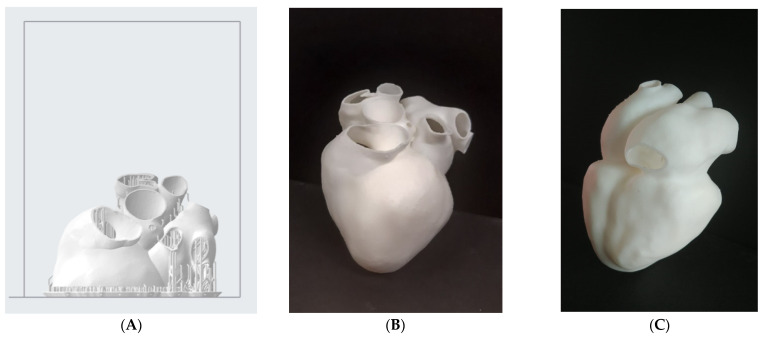
(**A**) “Superior” half of the hollow heart model disposed onto the printing platform (Preform 3.3); (**B**,**C**) the final print result, viewed according to two different orientations, as obtained after post-processing.

Afterwards, the hollow model was printed with the same rigid resin. Given the hollowed shape, support structures were needed even in the interior of the model. Since it is desirable to remove the support structures everywhere, it was not possible to print this model as a single body. Hence, it was split into 2 parts (the “superior” one, with the supporting structure, is shown in Figure 6A) to guarantee complete removal of internal supports. The global printing time for this hollow model was around 16 h and the post-processing phase was repeated as before. To ease the following scanner reacquisition work, the same model was also printed as one piece, with the full removal of internal supports not considered. The required printing time was essentially the same. This print result, after post-processing, can be seen in Figure 6B,C.

Starting from the hollow digital model, a test employing a flexible resin instead of the rigid one was also conducted. We chose the so-called “Elastic resin” by Formlabs, whose mechanical properties are shown in Table 3.

**Figure 7:**
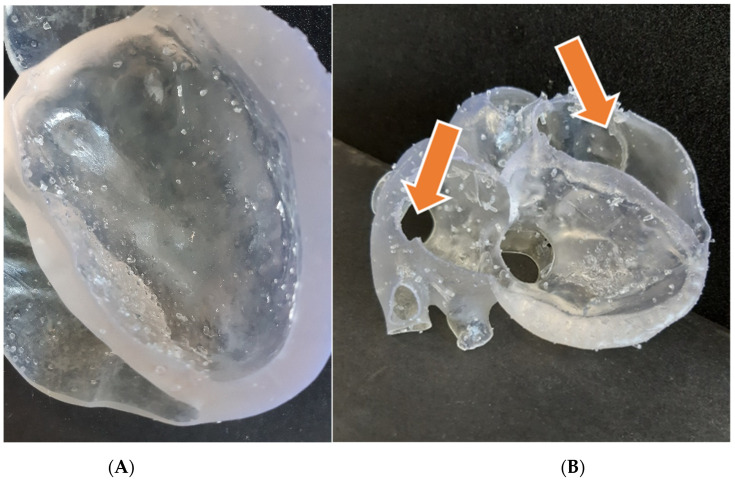
(**A**) Detail of the heart model (lower part) printed in flexible resin, in which residual touchpoints are clearly visible; (**B**) the corresponding upper part, broken in some points (see arrows) during support removal.

We exploited the previously reconstructed model, without further modifications, even if some changes had to be made in the preprint phase. Indeed, dealing with a rubber-like resin, the printing process is much more delicate, and a denser and well-organized supports net has to be applied. The global printing time for this model was around 36 h, much longer than the previous ones. The post-processing settings, however, were quite similar, as suggested by the material supplier. The resulting model is endowed with very good compliance, especially where the wall is thinner. The final appearance, beyond the transparency, is affected by the presence of residual support touchpoints (Figure 7A), which can be clearly appreciated to the touch and are more difficult to remove than in the previous cases. Moreover, due to the difficulties in this operation, the wall of the upper part broke in some points (Figure 7B) because of the need to enter in-depth with the flash cutter, trying to remove all the support structures. It is likely that this inconvenience could be mitigated by applying a thicker wall, while assigning the offset. Nevertheless, we concluded that if we wished to move to flexible resin prints, another printing technology would be desirable.

### 3.3. Dimensional Accuracy Evaluation

Besides the qualitative evaluation made so far, we evaluated dimensional accuracy with reference to the original STL models. To survey the printed objects, we used the *NextEngine* Ultra HD laser scanner, a multi-stripe triangulation-based laser scanner equipped with a rotating table. The measuring equipment was set up with a scanning distance of around 250 mm, “macro mode” setting and capture density set at 440 points/mm^2^. This setup allows the acquisition to reach up to 100 µm accuracy. Each object was surveyed with 12 scans around 360 degrees, i.e., one scan every 30 degrees. After a first round, a second one was performed, changing the orientation of the object on the rotating table in order for the scanner to see the whole object. Both the rigid models, i.e., the blood pool and the hollow heart, were scanned. For the hollow model, only the one-piece print was considered.

**Figure 8:**
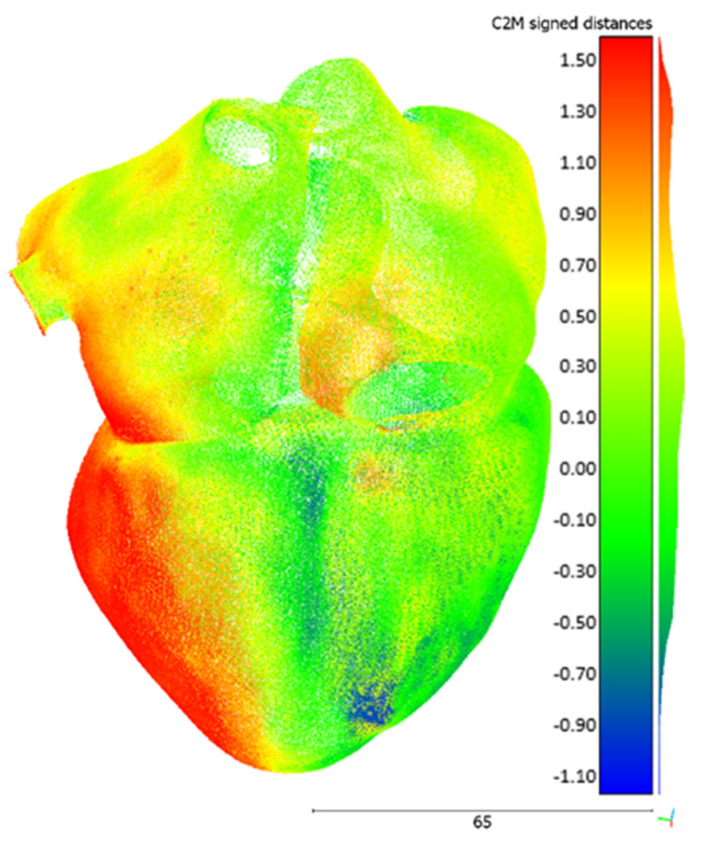
Hollow heart model comparison result in CloudCompare. Values are in millimeters.

The raw data were registered and post-processed in *ScanStudio* 2.0, the proprietary software of the scanner, and then exported as a point cloud. The alignment and the registration of the different scans ended up with an average error of 7 µm. The comparison between each point cloud and the respective STL file was performed in open-source software *CloudCompare* (GPL software), version 2.11. The reference mesh was the original STL, while the compared entity was the acquired point cloud. The tool computes the Euclidean distance between the two entities, searching, for each point of the compared cloud, the nearest triangle in the reference mesh. The graphical result for the hollow heart is shown in Figure 8. The contour map indicates the signed distance between the mesh and the acquired point cloud. As a comprehensive metric, the average and the standard deviation of the unsigned (i.e., the absolute value) distances for each comparison were calculated. Concerning the blood pool, the metrics are 0.19 mm and 0.14 mm for the average and the standard deviation, respectively. Regarding the hollow model, the same are 0.53 mm and 0.39 mm, respectively.

## 4. Conclusions and Discussion

In this study, accurate 3D anatomical models of the heart, both as a blood pool and as a hollow organ, were generated starting from a stack of CT scans. They were then employed to realize physical replicas by means of additive manufacturing.

As can be expected, the hollow model is slightly less accurate than the blood pool. Indeed, as the minimum wall thickness of the hollow model is around 1 mm, the accuracy of the printer is close to its critical value: Formlabs suggests 0.6 mm as the minimum thickness for unsupported thin walls. Furthermore, the wall in the bottom part of the model reaches more than 10 mm of thickness and this probably causes the barycenter of the object to fall out of the projection center of the build plane. This is likely the cause of the red area visible in Figure 8, representing a displacement extending from the original shape. This problem is not present for the other model. Besides this consideration, both models can be considered suitable for visual applications, such as education and communication. Further investigations are needed when it comes to applying these models to in vitro testing or any kind of quantitative assessment of the cardiovascular system.

A more sophisticated approach for accuracy evaluation would rely on the reacquisition of the printed models with a CT scanner, ideally the same employed for original CT acquisition. In this way, even the internal part of the heart could be evaluated by means of the overlay of reacquired model boundaries on the original scans. Even if surely more rigorous and robust, this method is slightly more elaborate and complex, requiring also the availability of a medical scanner.

A separate discussion is due for the flexible resin model. SLA technology did not turn out to be the best option for this kind of print. As we saw, a dense support structure is needed and, when supports are manually removed, small but visible spikes remain on the surface, affecting its final quality. As we observed, these flexible supports are practically much more difficult to remove and the risk of damaging the model is considerable. Moreover, only a limited set of distensible resins is nowadays available for Formlabs printers, so an accurate calibration of mechanical properties according to the specific application cannot be performed. In this case, material jetting technology may be the best option, as discussed in detail in [39], to print multi-material models and exploit soluble support structures. In this way, first of all, there is no longer the need to split the model into two parts to remove inner supports. Moreover, material blends, tuning the final shore hardness according to the specific needs, can be created starting from a wider set of available polymers. Here, the main obstacle remains the high cost, both of the printer and of the resins, which prevents most users from accessing this technology.

To extend the realism and the employability of our printed heart model, the introduction of valves, with sub-valvular apparatus, would also be very useful. They could be exploited, for example, to realize ad hoc training simulators for transcatheter repair or replacement procedures. The problem is that these structures are very difficult to identify and isolate from CTs. The association of CT images with ad hoc imaging techniques, such as 3D echocardiography, would allow easy capturing also of valve leaflets. Even the introduction of an accurate replica of the *chordae tendineae* would be a demanding challenge, both in terms of segmentation and physical reproduction.

Another important point to focus on is the time required to generate this kind of model. Segmentation is a very time-consuming and tedious activity, subject to intra- and inter-observer variability, and requires dedicated expert operators [10], especially in the case of complex anatomies. To give an idea, the complete process to obtain the hollow heart from the raw images to the final smoothed model, without considering the printing phase, took us some tens of hours. Thus, greater automation of the image segmentation and geometry modeling techniques would be desirable to reduce the amount of time that this kind of process still requires [9]. Several attempts in automating the segmentation process can be found in the literature [40], but they usually provide rough results, and, regardless, human intervention, even if limited, is always necessary. A common limitation of currently diffused artificial intelligence-based segmentation approaches is, as mentioned before, their inability to tackle previously unseen samples (e.g., data from new scanners or abnormal and pathological cases not included in the training set). This prevents them from being deployed in real-world applications and so diminishes their impact in clinical workflows [8]. This is especially true for the whole heart, given the great complexity of the anatomy and its great variability, for which we are still somewhat far from a fully automated and robust segmentation approach.

One last point is as follows: the accuracy and reproducibility of the whole workflow from which 3D-printed models are obtained should be tested [12] considering a sufficient poll of subjects. Some partial studies in this sense have been conducted (see, e.g., [41]), but they focus on bones, while valuable soft tissue studies are lacking in the literature to our knowledge. Several factors can influence the model accuracy during the manufacturing process, but surely the most important elements are the segmentation and model post-processing phases, besides the print itself. Systematic assessment of intra-operator and inter-operator variability is an important future target. Standardization of the source image data acquisition and post-processing techniques will assist in this objective [12].

Once these crucial questions are effectively tackled, we are convinced that this technology will become widespread also in operative environments. Given the broad spectrum of anatomic variations and pathologies, 3D printing could potentially be a game-changer in cardiology, particularly in challenging anatomies and rare pathologies, facilitating procedural planning, optimal sizing and simulation. It could significantly improve the treatment of cardiovascular diseases, in the direction of patient-specific care, offering complementary engineering tools for this group of clinicians.

## Figures and Tables

**Table 1:** Strengths and weaknesses of widespread printing technologies, potentially suitable for employment in cardiovascular 3DP.

3DP Technology	Employed Materials	Producer (Example)	Spatial Resolution	Costs (Printer + Material)	Printing Time	Print Volume	Mono-/Multi- Material	Further Notes
**Fused Deposition Modeling (FDM)**	Thermoplastic filaments	Ultimaker	Generally quite low	Relatively cheap	Long	Limited	Multi-	Anisotropy Wide variety of materials
**Stereolithography (SLA)**	Photo-sensitive resin	Formlabs	Very good (up to 0.025 mm)	Relatively cheap	Long	Limited	Mono-	Extensive post-processing
**Selective Laser Sintering (SLS)**	Powdered polymers	EOS	Good (up to 0.060 mm)	Expensive	Very long (heating and cooling phases)	Large	Mono-	Complex machine preparation Safety issues
**Material jetting**	Photo-polymers	Stratasys	Excellent (up to 0.014 mm)	Very expensive	Shorter	Large	Multi-	High printer encumbrance

**Table 2:** Mechanical properties of the employed rigid resin (White V4), as declared by the producer. Source: Formlabs Materials Library.

	Green	Post-Cured	Method
**Ultimate tensile strength**	38 MPa	65 MPa	ASTM D 638-10
**Tensile modulus**	1.6 GPa	2.8 GPa	ASTM D 638-10
**Elongation at break**	12%	6%	ASTM D 638-10
**Notched IZOD**	16 J/m	25 J/m	ASTM D 638-10

**Table 3:** Mechanical properties of the employed “Elastic resin”, as declared by the producer. Source: Formlabs Materials Library.

	Green	Post-Cured	Method
**Ultimate tensile strength**	1.61 MPa	3.23 MPa	ASTM D 412-06
**Elongation at break**	100%	160%	ASTM D 412-06
**Tear strength**	8.9 kN/m	19.1 kN/m	ASTM D 624-00
**Shore hardness**	40 A	50 A	ASTM 2240

## Data Availability

Not applicable.

## Supplementary Materials

The following are available online at https://www.mdpi.com/article/10.3390/bioengineering8100130/s1, Heart_model.stl: 3D reconstruction of the hollow heart model presented in the work.
